# Correction: Construction of isostructural hydrogen-bonded organic frameworks: limitations and possibilities of pore expansion

**DOI:** 10.1039/d2sc90241a

**Published:** 2022-11-29

**Authors:** Yuto Suzuki, Mario Gutiérrez, Senri Tanaka, Eduardo Gomez, Norimitsu Tohnai, Nobuhiro Yasuda, Nobuyuki Matubayasi, Abderrazzak Douhal, Ichiro Hisaki

**Affiliations:** Division of Chemistry, Graduate School of Engineering Science, Osaka University 1-3 Machikaneyama Toyonaka Osaka 560-8531 Japan hisaki@chem.es.osaka-u.ac.jp; Departamento de Química Física, Facultad de Ciencias Ambientales y Bioquímica, INAMOL, Universidad de Castilla-La Mancha Avenida Carlos III, S/N 45071 Toledo Spain Abderrazzak.Douhal@uclm.es; Division of Chemical Engineering, Graduate School of Engineering Science, Osaka University 1-3 Machikaneyama Toyonaka Osaka 560-8531 Japan nobuyuki@cheng.es.osaka-u.ac.jp; Division of Applied Chemistry, Graduate School of Engineering, Osaka University 2-1 Yamadaoka Suita Osaka 565-7891 Japan; JASRI 1-1-1 Kouto, Sayo-cho Sayo-gun Hyogo 679-5198 Japan

## Abstract

Correction for ‘Construction of isostructural hydrogen-bonded organic frameworks: limitations and possibilities of pore expansion’ by Yuto Suzuki *et al.*, *Chem. Sci.*, 2021, **12**, 9607–9618, https://doi.org/10.1039/D1SC02690A.

The authors regret that Table 2 of the original article requires correction. On page 9616 of the original article, the first line of Table 2 ‘periodicity of the framework’ is incorrect. The amended version of Table 2 is shown below:

**Table:** Summary of the structural features and properties of the four isostructural HOFs based on HAT derivatives

	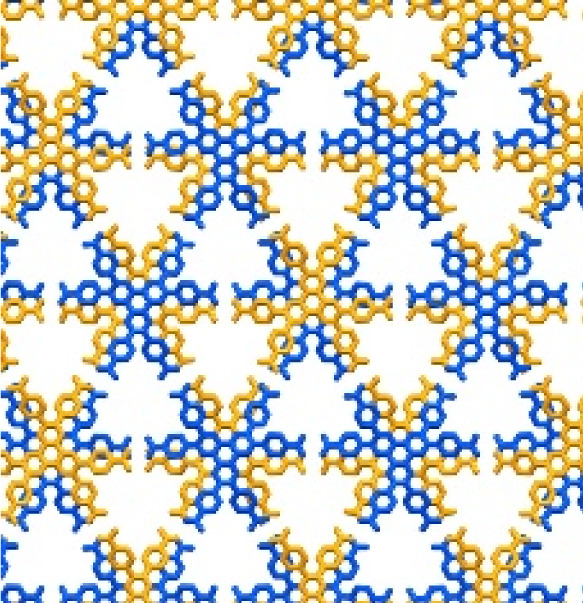	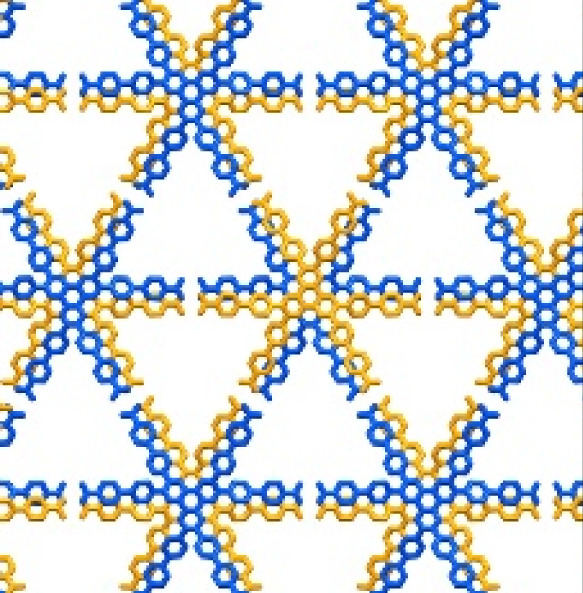	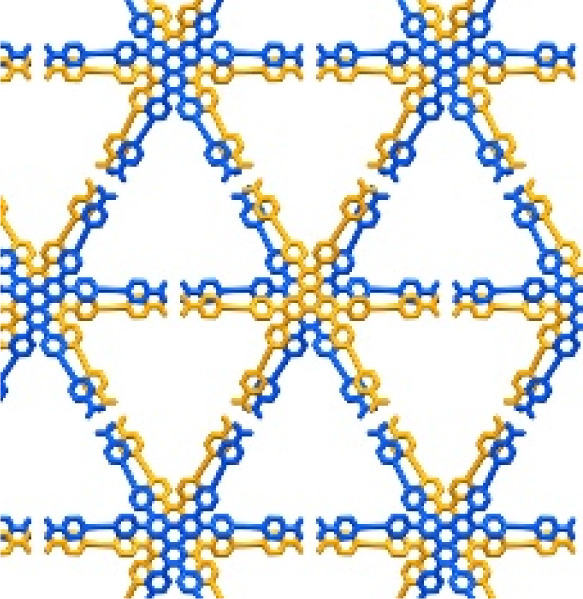	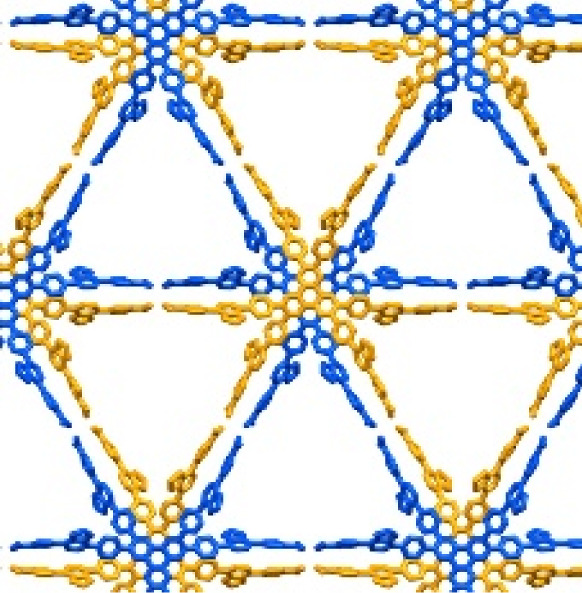
	CPHAT-1	CBPHAT-1	TolHAT-1	ThiaHAT-1
Periodicity of the framework/Å	21.48	29.75	34.40	38.01
RMSD of the HAT core plane/Å	0.267	0.205	0.215	0.229
Stacking distance/Å	3.59	3.57	3.49	3.49
Torsion angle of arms/°	22.5	22.1	23.5	24.5
Number of interpenetrations	4	6	8	8
Height of the channel aperture/Å	6.4	14.5	19.2	18.0
Void ratio	0.31	0.45	0.55	0.48
Pore width based on NLDFT/Å	—a	12.4	16.6	15.5
BET surface area/m^2^ g^−1^	649	1288	440	1394
N_2_ uptake/mL (STP) g^−1^	21.39	361.7	155.2	415.7
CO_2_ uptake/mL (STP) g^−1^	137.4	304.5	168.6	313.9
Decomposition temp./°C	339	307	190	305
Ref.	Ref. 36	Ref. 37	This work	This work

a Not determined.

The Royal Society of Chemistry apologises for these errors and any consequent inconvenience to authors and readers.

## Supplementary Material

